# A Security-Enhanced Image Communication Scheme Using Cellular Neural Network

**DOI:** 10.3390/e23081000

**Published:** 2021-07-31

**Authors:** Heping Wen, Jiajun Xu, Yunlong Liao, Ruiting Chen, Danze Shen, Lifei Wen, Yulin Shi, Qin Lin, Zhonghao Liang, Sihang Zhang, Yuxuan Liu, Ailin Huo, Tong Li, Chang Cai, Jiaqian Wen, Chongfu Zhang

**Affiliations:** 1 Zhongshan Institute, University of Electronic Science and Technology of China, Zhongshan 528402, China; wenheping@uestc.edu.cn (H.W.); xujiajun@stu.zsc.edu.cn (J.X.); teamoyan@stu.zsc.edu.cn (Y.L.); ruitingchen@stu.zsc.edu.cn (R.C.); shendanze@stu.zsc.edu.cn (D.S.); wenlifei@stu.zsc.edu.cn (L.W.); shiyulin@stu.zsc.edu.cn (Y.S.); liqin@stu.zsc.edu.cn (Q.L.); zhonghaoliang@stu.zsc.edu.cn (Z.L.); sihangzhang@stu.zsc.edu.cn (S.Z.); liuyuxuan@stu.zsc.edu.cn (Y.L.); ailinhuo@stu.zsc.edu.cn (A.H.); litong@stu.zsc.edu.cn (T.L.); caichang@stu.zsc.edu.cn (C.C.); jiaqianwen@stu.zsc.edu.cn (J.W.); 2 School of Information and Communication Engineering, University of Electronic Science and Technology of China, Chengdu 611731, China; 3 Guangdong Provincial Key Laboratory of Information Security Technology, Guangzhou 510006, China

**Keywords:** secure communication, image encryption, chaos, cellular neural network

## Abstract

In the current network and big data environment, the secure transmission of digital images is facing huge challenges. The use of some methodologies in artificial intelligence to enhance its security is extremely cutting-edge and also a development trend. To this end, this paper proposes a security-enhanced image communication scheme based on cellular neural network (CNN) under cryptanalysis. First, the complex characteristics of CNN are used to create pseudorandom sequences for image encryption. Then, a plain image is sequentially confused, permuted and diffused to get the cipher image by these CNN-based sequences. Based on cryptanalysis theory, a security-enhanced algorithm structure and relevant steps are detailed. Theoretical analysis and experimental results both demonstrate its safety performance. Moreover, the structure of image cipher can effectively resist various common attacks in cryptography. Therefore, the image communication scheme based on CNN proposed in this paper is a competitive security technology method.

## 1. Introduction

With the rapid development of cloud computing, big data, blockchain and other emerging technologies, the privacy and sharing of messages provides convenience for people in their work and daily lives [1,2,3,4]. However, the convenience also threatens the security of cyberspace [5,6,7,8]. In particular, as a significant transmission medium, digital images may include a lot of personal privacy, confidential information and other important data, so their privacy protection gets more attention [9,10,11,12]. Encryption technology is a common means to assure the security of digital images, and has been widely used in various fields of digital image security [13,14,15,16,17]. Currently, there exist many mature block encryption schemes that are widely used in text encryption and these schemes have brilliant effects [18,19]. Nevertheless, due to the uniqueness of the image, such as being two-dimensional, redundancy and a strong correlation of two adjacent pixels, traditional text encryption faces severe challenges [20,21,22]. Moreover, the problem of real-time transmission should be considered in image encryption to improve the communication performance [9,23,24]. Therefore, it is quite necessary to study the new technologies and methods of image encryption.

In current international studies, digital image encryption is a research hotspot [25,26,27]. Various mechanisms and methods are introduced to enhance the security of algorithms [28,29]. In 2015, the authors of [16] proposed a multibiometric template protection scheme based on fuzzy commitment and a chaos-based system, as well as a security analysis method of unimodal biometrics leakage. The chaos-based system is used to encrypt the dual iris feature vectors. The experimental results show that the security of BCH ECC (1,023,123,170) based on multibiometrics template is improved from 80.53 bits to 167.80 bits. In 2017, the authors of [30] designed a special image encryption scheme based on the second-order Henon mapping hyperchaos and the fifth-order CNN. Experimental results show that the scheme features high security and is suitable to spread in the network. At the same time, in [31] a new image encryption method was proposed, based on the biological DNA sequences operation and the third-order CNN. The method could effectively enhance the plaintext sensitivity and features large key space and high security. In 2019, the authors of [17] proposed a new privacy protection encryption mechanism for medical systems based on the Internet of Things. Experimental results show that the encryption mechanism is robust and effective to protect the privacy of patients. In 2020, Zhang and Zhang [32] used the Chen chaos-based system and two-dimensional logistic mapping to propose a multi-image encryption system based on bitplane and chaos. The experiment also proved its high efficiency. At the same time, in [15] a new and effective color image cryptosystem was proposed. The experimental results show that the cryptosystem has high security efficiency and can be effectively applied to the IoHT framework of secure medical image transmission. In summary, more and more theories and technological achievements have been made in digital image encryption. However, in current studies, most digital images are regarded as a two-dimensional matrix to encrypt, meaning that only the spatial domain is processed [6,33,34,35]. However, two defects were exposed: (1) Some encryption algorithms have security flaws and are not associated with plaintext, so it is difficult for them to resist chosen-plaintext attack (CPA); (2) The cost of attacking the encryption algorithm is relatively low because chaos-based systems are relatively simple.

Aimed at solving the existing problems, we put forward a digital image encryption algorithm based on CNN in this paper. On the one hand, a CNN chaos-based system is selected to generate a chaos-based key sequence. The CNN chaos-based system has more complex behavioral characteristics, so it has better security performance than other encryption systems. On the other hand, the scheme adopts the security mechanism of generating a chaos-based key sequence by plaintext correlation. Therefore, compared with other encryption schemes based on a CNN chaos-based system, it effectively enhances the ability to resist CPA. Theoretical analysis and experimental results show that the proposed algorithm can effectively enhance the confusion, diffusion and avalanche effect of encryption. Therefore, the image encryption algorithm based on CNN is reliable.

## 2. Correlation Theory

The idea of a cellular neural network (CNN) was conceived by Chua and Yang in 1988 [34]. The basic units of CNN are called cells, and each cell is a nonlinear first-order circuit which is composed of a linear resistor, a linear capacitor and a voltage-controlled current source [36,37].

In order to make the mathematical model of CNN more comprehensible, a simplified CNN cell model is adopted:

(1) $$\frac{dx_{j}}{dt}=-x_{j}+A_{j}p_{j}+G_{o}+G_{s}+I_{j}$$

where *j* is used as a cell marker, $x_{j}$ represents the state variable, $A_{j}$ represents a constant number, $I_{j}$ represents the threshold value, $G_{s}$ and $G_{o}$ separately represent the linear combination of the state variables of the cell and the output value of the connecting cell, and $p_{j}$ represents the output of the cell.

**Figure 1:**
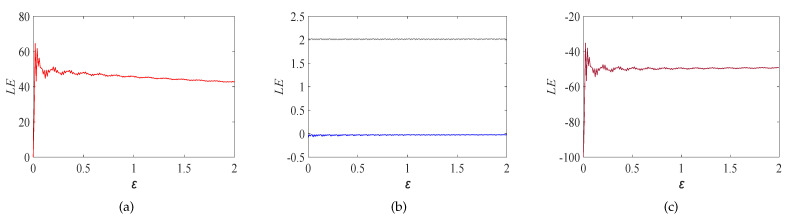
Lyapunov exponents spectrum. The exponents tend to 42.8487, 2.0230 and −0.0230, and −49.0391, as can be seen in (**a**–**c**), respectively.

**Figure 2:**
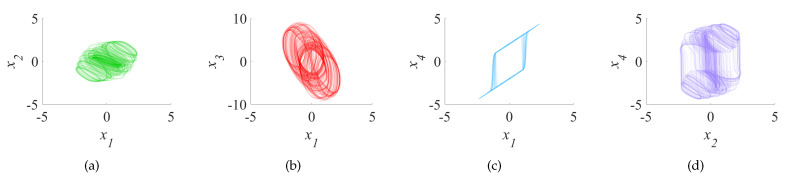

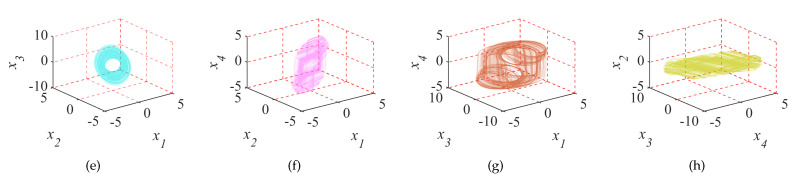
Chaos-based attractors generated by the fourth-order CNN: (**a**) $x_{1},x_{2}$; (**b**) $x_{1},x_{3}$; (**c**) $x_{1},x_{4}$; (**d**) $x_{2},x_{4}$; (**e**) $x_{1},x_{2},x_{3}$; (**f**) $x_{1},x_{2},x_{4}$; (**g**) $x_{1},x_{3},x_{4}$; (**h**) $x_{4},x_{3},x_{2}$.

The fourth-order fully interconnected CNN equation can be defined as follows:

(2) $$\left\{\begin{array}{c} \frac{dx_{j}}{dt}=-x_{j}+A_{j}p_{j}+\sum_{k=1;k\neq j}^{4}A_{jk}p_{j}+\sum_{k=1}^{4}S_{jk}x_{k}+I_{j}\\ p_{j}=0.5\left|x_{j}+1\right|-0.5\left|x_{j}-1\right| \end{array}\right.$$

where *S* represents a matrix of j×k, $A_{j}$ and $I_{j}$ both represent a matrix of j×1, $A_{jk}=0(j\neq k,j=$1,2,3,4;k=1,2,3,4) and it can be described by the equation of state in Equation (2) [38]:

(3) $$\left\{\begin{array}{cc} \;\frac{dx_{1}}{dt} & =-x_{3}-\varepsilon x_{4}\\ \frac{dx_{2}}{dt} & =2x_{2}+x_{3}\\ \frac{dx_{3}}{dt} & =14x_{1}-14x_{2}\\ \frac{dx_{4}}{dt} & =200p_{4}+100x_{1}-100x_{4} \end{array}\;\right.$$

where ε is the control parameter of the CNN model, which can control the size and quantity of Lyapunov exponents, and the range of values for ε is 0 to 2. At this moment, the system is in a chaos-based state, and four aperiodic chaos-based sequences can be generated from it, which are very sensitive to the initial conditions $x_{1}\left(0\right),x_{2}\left(0\right),x_{3}\left(0\right)$ and $x_{4}\left(0\right)$. By calculating the Lyapunov exponents of Equation (3), it can be seen that the Lyapunov exponents of the four chaos-based sequences tend to 42.8487, 2.0230, −0.0230 and −49.0391, respectively, two of which are positive. Therefore, the CNN model is a hyperchaotic system, and the Lyapunov exponents are shown in Figure 1. When the initial values of $x_{1}\left(0\right)$, $x_{2}\left(0\right)$, $x_{3}\left(0\right)$ and $x_{4}\left(0\right)$ are 0.2, 0.2, 0.2 and 0.2, respectively, we use the fourth-order Runge–Kutta algorithm with the step size of *h* = 0.005 to get the two-dimensional chaos-based attractor, as shown in Figure 2a–d and the three-dimensional chaos-based attractor, as shown in Figure 2e–h.

## 3. The Proposed Encryption Algorithm

The encryption algorithm of chaos-based image usually adopts the classical structure “permutation–diffusion” [39,40]. However, due to the lack of security, a chaos-based image encryption algorithm based on a “confusion–permutation–diffusion” structure is proposed in this paper [35].

**Figure 3:**
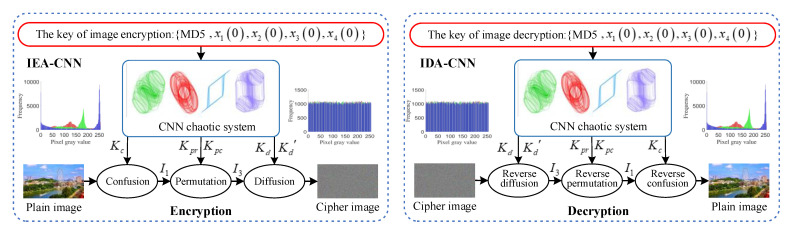
Principle and mechanism of image encryption and decryption.

The encryption and decryption processes are shown in Figure 3. IEA-CNN represents the image encryption algorithm based on a cellular neural network, IDA-CNN represents the image decryption algorithm based on a cellular neural network. In order to enhance the ability to resist CPA, the image encryption system of this paper adopts the security mechanisms of chaos-based key sequences produced by plaintext association and ciphertext feedback diffusion encryption. The specific steps of the encryption algorithm are given as follows:

**Step 1:** *Preprocessing Sequences*

**Figure 4:**
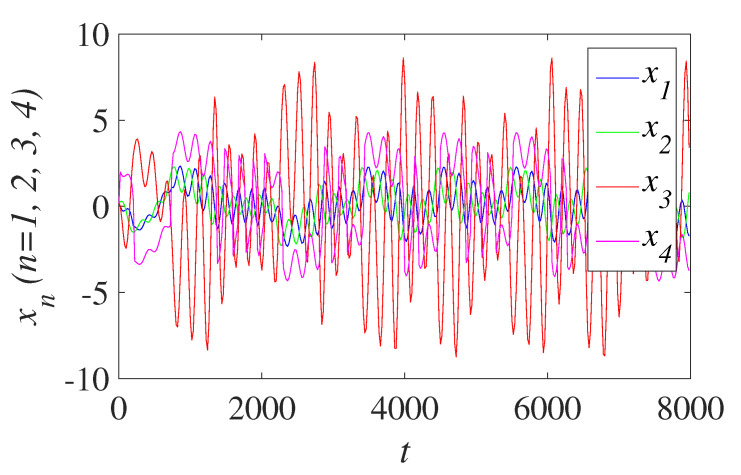
Sequence diagram of the fourth-order CNN.

The secret key of the image encryption algorithm contains the Message-Digest Algorithm 5 (MD5) value of plain image, the initial value of the fourth-order CNN and the controlling parameters. The MD5 can be used to disturb the initial value key parameters of CNN chaos; so that the key sequence changes with different plain images, the specific treatment methods are calculated using the following formulas:

(4) $$\left\{\begin{array}{c} x_{1}^{\prime }\left(0\right)=x_{1}\left(0\right)+\left(m_{1}\;⊕m_{2}\;⊕m_{3}\;⊕m_{4}\;\right)/256\\ x_{2}^{\prime }\left(0\right)=x_{2}\left(0\right)+\left(m_{5}\;⊕m_{6}\;⊕m_{7}\;⊕m_{8}\;\right)/256\\ x_{3}^{\prime }\left(0\right)=x_{3}\left(0\right)+\left(m_{9}\;⊕m_{10}⊕m_{11}⊕m_{12}\right)/256\\ x_{4}^{\prime }\left(0\right)=x_{4}\left(0\right)+\left(m_{13}⊕m_{14}⊕m_{15}⊕m_{16}\right)/256 \end{array}\right.$$

where ⊕ is bitwise XOR operation, $x_{1}\left(0\right),x_{2}\left(0\right),x_{3}\left(0\right)$ and $x_{4}\left(0\right)$ are the initial values of the fourth-order CNN key parameters; ${x_{1}}^{\prime }\left(0\right),{x_{2}}^{\prime }\left(0\right),{x_{3}}^{\prime }\left(0\right)$ and ${x_{4}}^{\prime }\left(0\right)$ are the initial values updated after the disturbance from MD5. Obviously, the new initial values will change with the different plain images. Then, a preprocessing operation is adopted for the chaos-based sequences. The generating methods of obfuscated sequences are shown as follows:

(5) $$\left\{\begin{array}{c} real\_X=\left[x_{1};x_{2};x_{3};x_{4}\right]\\ {K_{c}}^{\prime }=floor\left(\;\;\mathrm{mod}\;\;\left(real\_X\times 10^{10},256\right)\right)\\ K_{c}=reshape\left({K_{c}}^{\prime },H,W\right) \end{array}\right.$$

where real_X is composed of four sequences produced by the fourth-order CNN chaos-based system. The sequences diagram of four sequences generated by chaos-based mapping of the fourth-order CNN is shown in Figure 4. The size of $K_{c}$ is equal to H×W, *H* and *W* are pixel rows and pixel columns of the plain images for image confusion. The generating method of permutation sequences is shown as follows:

(6) $$\left\{\begin{array}{c} seq\_H=x_{2}\left(1,1:H\right)\\ seq\_W=x_{3}\left(2,1:8\times W\right)\\ \left[value_{1},K_{pr}\right]=sort\left(seq\_H\right)\\ \left[value_{2},K_{pc}\right]=sort\left(seq\_W\right) \end{array}\right.$$

where sort is the sorting function of array elements; $x_{2}$ represents a two-dimensional sequence of real_X; $x_{3}$ represents the three-dimensional sequence of real_X; seq_H represents the chaos-based sequence of length *H* extracted from $x_{2}$; real_W represents the chaos-based sequence of length 8×W extracted from $x_{3}$; $K_{pr}$ means that the pixel row is generated by the sorting function and the length is *H*; $K_{pc}$ means that the pixel column is generated by the sorting function and the length is 8×W; $value_{1}$ and $value_{2}$ are the sorted chaos-based sequence values.

The generating method of diffusion sequences is shown as follows:

(7) $$\left\{\begin{array}{c} K_{d}=mod\left(floor\left(\left[x_{1},x_{3},x_{2},x_{4}\right]\times 10^{5}\right),256\right)\\ {K_{d}}^{\prime }=mod\left(floor\left(\left[x_{3},x_{4},x_{1},x_{2}\right]\times 10^{5}\right),256\right) \end{array}\right.$$

where the lengths of $K_{d}$ and ${K_{d}}^{\prime }$ are H×W, and the key sequences of $K_{d}$ and ${K_{d}}^{\prime }$ are used for diffusion.

**Step 2:** *Confusion*

The key sequence $K_{c}$ is used to obfuscate the plain image *P*. The image can be visualized and hidden to get the obfuscated image $I_{1}$, the method is shown as follows:

(8) $$\begin{array}{cc} I_{1}\left(i\right) & =K_{c}\left(i\right)⊕P\left(i\right),i=\left(1,2,\cdots ,H\times W\right) \end{array}$$

**Step 3:** *Permutation*

The key sequences $K_{pr}\left(i\right)$ and $K_{pc}\left(j\right)$ are used to replace the pixels in $I_{1}$ to get $I_{3}$, the method is shown as follows:

(9) $$\begin{array}{c} \left\{\begin{array}{c} I_{2}=\mathrm{sw}ap\left(I_{1}\left(:,K_{pc}\left(i\right)\right),I_{1}\left(:,i\right)\right)\\ I_{3}=\mathrm{sw}ap\left(I_{2}\left(K_{pr}\left(j\right),:\right),I_{2}\left(j,:\right)\right) \end{array}\right. \end{array}$$

where swap function is used to swap the values of two pixels. The number of bit level rows is equal to the number of pixel level rows, and the number of bit level columns is equal to 8 times the number of pixel level columns, thus, i=1,2,⋯,H and j=1,2,⋯,8×W. $I_{2}$ and $I_{3}$ are the images after double bit column transform and row transform permutation, respectively.

**Step 4:** *Diffusion*

All the ciphertext pixels in $I_{3}$ are diffused dynamically. $K_{d}$ and $K_{d}^{\prime }$ are used for the image diffusion operation to generate the final ciphertext image C.

The first ciphertext pixel C(1) is generated, and the diffusion encryption equation is shown as follows:

(10) $$\left\{\begin{array}{c} C\left(1\right)=I_{3}\left(1\right)⊕K_{d}\left(1\right)⊕\left(sum\left(1\right)\dot{+}{K_{d}}^{\prime }\left(1\right)\right)\\ sum\left(1\right)=\sum_{i=1}^{L}I_{3}\left(i\right) \end{array}\right.$$

where the operator $\dot{+}$ can be defined as $a\dot{+}b\overset{\Delta }{\;=}\;\mathrm{mod}\;\left(a+b,256\right)$, $I_{3}\left(1\right)$ is the first pixel of the permutation image $I_{3},K_{d}\left(1\right)$ and ${K_{d}}^{\prime }\left(1\right)$ are the first element of the diffusion encryption sequences, and sum(1) represents the sum of all pixels of the permutation image $I_{3}$.

Then ciphertext pixel C(i) is produced and its diffusion formula is shown as follows:

(11) $$\left\{\begin{array}{c} C\left(i\right)=I_{3}\left(i\right)⊕\left(C\left(i-1\right)\dot{+}K_{d}\left(i\right)\right)⊕\left(sum\left(i\right)\dot{+}{K_{d}}^{\prime }\left(i\right)\right)\\ sum\left(i\right)=sum\left(i-1\right)-I_{3}\left(i\right) \end{array}\right.$$

where i=2,3,⋯,L and the *i* represents the *i*th pixel of the permutation image $I_{3}$. C(i−1) is the (i−1)th ciphertext pixel. sum(i) is the sum of the (L−i+1) pixels of the permutation image $I_{3}$. According to Equation (11), starting from the second ciphertext pixel C(2), the cipher image *C* is generated by computing iteratively C(i), *i* in $\left\{1,2,\cdots ,L\right\}$, until the *L*th ciphertext C(L) is generated.

Decryption is the inverse process of encryption, whose process is first confusion, then permutation, and finally diffusion. While the decryption process is to first reverse diffuse the encrypted image, then reverse permutate the reverse diffuse image, and finally reverse confuse the reverse permutation image to get the decrypted image. When the decryption key and the encryption key are matched, the image can be restored correctly. However, when the decryption key is not equal to the encryption key, even if there is a small error, the correct image cannot be decrypted.

## 4. Experimental Verification and Discussion

In the analysis of the experimental results, we use MATLAB 2020b to simulate and validate the proposed image encryption system which is executed on a PC with Windows 10 64 bit operating system, Intel (R) Core (TM) i7-8250 CPU @ 1.60 GHz 1.80 GHz processor and 8 GB memory. In order to prove the effectiveness and practicability of the proposed image encryption scheme, we selected the images from “USC-SIPI Image Database” and “Ground Truth Database” as the test images [41,42].

### 4.1. Key Space Analysis

In the encryption system, the range of valid value of key can be expressed by key space. The image encryption algorithm designed in this paper uses a fourth-order CNN system and the secret key parameters involved are the initial values of the fourth-order CNN chaos-based system $x_{1}\left(0\right),x_{2}\left(0\right),x_{3}\left(0\right),x_{4}\left(0\right)$. Because the computer precision used in experimental simulation is $10^{-15}$, the size of this part of encryption system key space is ${\left(10^{15}\right)}^{4}=10^{60}\approx 2^{199}$. Considering that MD5 of 128 bits can also be used as part of the secret key, the total secret key space $2^{327}$ and the encryption system can resist the exhaustive attack effectively [43,44].

### 4.2. Nist 800-22 Test

The NIST 800-22 test is an internationally recognized random number test. It consists of 16 different tests. As long as the 16 test results are greater than or equal to 0.001, the random array can be considered to be qualified. In this test, we divide the generated 3,000,000 bits of byte stream data into 10 segments of 300,000 bits. The $K_{c},K_{pr},K_{pc},K_{d}$ and $K_{d}^{\prime }$ sequences needed in encryption passed the test successfully, and the test results of the $K_{d}^{\prime }$ sequence are shown in Table 1. The experimental results show that the random numbers generated by our algorithm fully conform to the international standard, and have strong randomness.

### 4.3. Histogram Analysis

**Figure 5:**
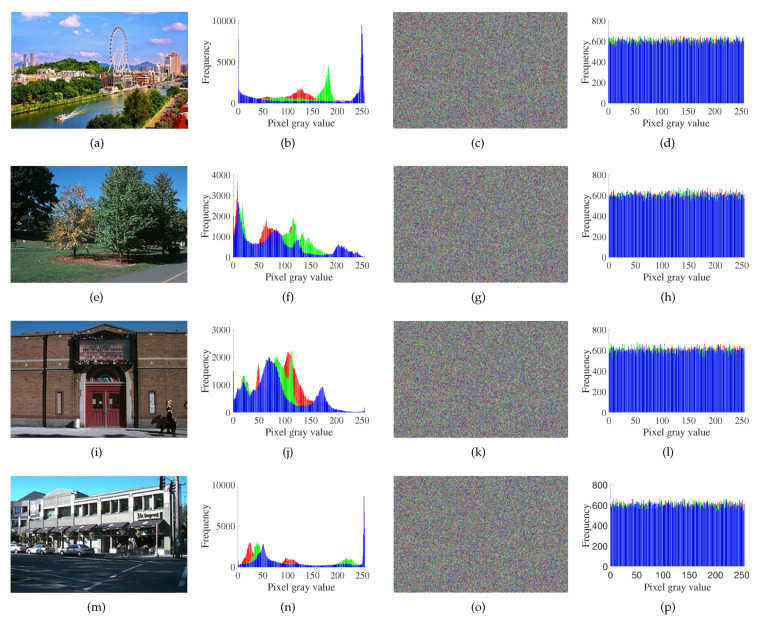
The histograms of images before and after encryption: (**a**) plain image of “Zhong shan”; (**b**) histogram of the plain image of “Zhong shan”; (**c**) cipher image of “Zhong shan”; (**d**) histogram of the cipher image of “Zhong shan”; (**e**) plain image of “Greenlake10”; (**f**) histogram of the plain image of “Greenlake10”; (**g**) cipher image of “Greenlake10”; (**h**) histogram of the cipher image of “Greenlake10”; (**i**) plain image of “Greenlake13”; (**j**) histogram of the plain image of “Greenlake13”; (**k**) cipher image of “Greenlake13”; (**l**) histogram of the cipher image of “Greenlake13”; (**m**) plain image of “Greenlake47”; (**n**) histogram of the plain image of “Greenlake47”; (**o**) cipher image of “Greenlake47”; (**p**) histogram of cipher image of “Greenlake47”.

There are three channels—R, G and B—in color images; the abscissa of the histogram containing these three channels reflects the statistical characteristics of the distribution of every pixel [45,46]. Different plain images and cipher images, as well as their relevant histograms, are shown in Figure 5. The experimental results show that the pixel values of the R, G and B channels of color cipher image are almost uniformly distributed, so the influence of statistical analysis is greatly eliminated [47,48].

### 4.4. Correlation Analysis

For the plain image, the correlation between adjacent pixels is strong [49,50]. Gray value of a pixel tends to be close to the gray values of its adjacent pixels. Therefore, the attacker can speculate about the gray value of a pixel from the gray value of its adjacent pixels [51,52]. An encryption system with good performance should satisfy the requirement that adjacent pixels of cipher image have low correlation coefficients to each other in order to resist the statistical attack. Correlation coefficients are commonly used to measure the correlation of two pixels and the calculations of it are defined as [53,54]:

(12) $$\left\{\begin{array}{c} E\left(x\right)=\frac{1}{N}\sum_{i=1}^{N}x_{i}\\ D\left(x\right)=\frac{1}{N}\sum_{i=1}^{N}{\left(x_{i}-E\left(x\right)\right)}^{2}\\ \mathrm{cov}\left(x,y\right)=\frac{1}{N}\sum_{i=1}^{N}\left(x_{i}-E\left(x\right)\right)\left(y_{i}-E\left(y\right)\right)\\ \gamma_{xy}=\frac{\mathrm{cov}(x,y)}{\sqrt{D(x)}\times \sqrt{D(y)}} \end{array}\right.$$

where the gray value of every pixel is represented by *x* and *y*, while E(x) represents the mean value, D(x) represents the variance, cov(x,y) represents the covariance and $\gamma_{xy}$ represents the correlation coefficients.

**Figure 6:**
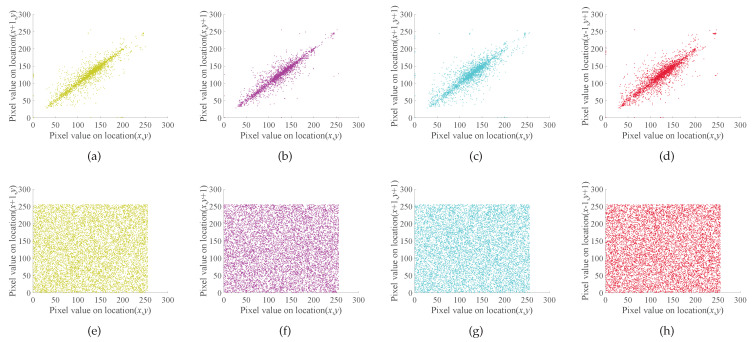
Correlation coefficients distribution map of plain image and cipher image of “7.1.02.tiff”: (**a**) “7.1.02.tiff” plain image horizontal correlation; (**b**) “7.1.02.tiff” plain image is vertical correlation; (**c**) “7.1.02.tiff” plain image diagonal correlation; (**d**) “7.1.02.tiff” plain image against angular direction correlation; (**e**) “7.1.02.tiff” cipher image horizontal correlation; (**f**) “7.1.02.tiff” cipher image vertical correlation; (**g**) “7.1.02.tiff” cipher image diagonal correlation; (**h**) “7.1.02.tiff” cipher image inverse diagonal correlation.

The correlation coefficients before and after encryption of the selected image are shown in Table 2 where “Anti-Diag”. represents the correlation coefficient in the anti-diagonal direction. Figure 6 shows the correlation of plain image and cipher image in horizontal, vertical, diagonal and anti-diagonal directions. It can be seen that there is no obvious correlation between adjacent pixels of a cipher image. Therefore, the cipher images encrypted by the algorithm designed in this paper have high security and can resist the statistical analysis [55].

### 4.5. Sensitivity Analysis

**Figure 7:**
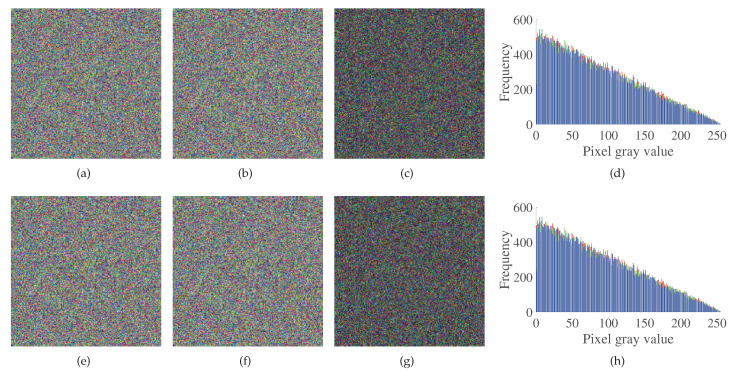
The key sensitivity test: (**a**) $x_{1}\left(0\right),x_{2}\left(0\right),x_{3}\left(0\right),x_{4}\left(0\right)$; (**b**) $x_{1}\left(0\right)+10^{-15},x_{2}\left(0\right),x_{3}\left(0\right),x_{4}\left(0\right)$; (**c**) Difference image after key perturbation; (**d**) Difference histogram after key perturbation; (**e**) $x_{1}\left(0\right),x_{2}\left(0\right),x_{3}\left(0\right),x_{4}\left(0\right)$; (**f**) $x_{1}\left(0\right),x_{2}\left(0\right)+10^{-15},x_{3}\left(0\right),x_{4}\left(0\right)$; (**g**) Difference image after key perturbation; (**h**) Difference histogram after key perturbation.

**Figure 8:**
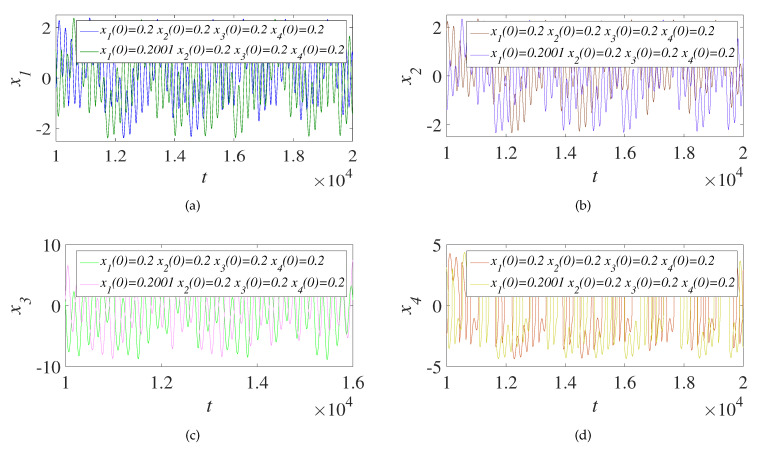
Comparison of four sequences (**a**–**d**) before and after key $x_{1}\left(0\right)$ perturbation.

Key sensitivity is an essential indicator of the security of the encryption system. It represents the difference in the decryption results when the same cipher image is decrypted with slightly different keys. For the sake of detecting the susceptibility of the scheme to the key, the first three sequences generated by the initial key are superimposed and combined into a color map, and the minimum precision of $x_{1}\left(0\right)$ is $10^{-15}$. The initial key $x_{1}\left(0\right)$ is perturbed with the minimum precision to generate four new sequences, and the first three new sequences are superimposed and combined into a new color map. The two color images are differentiated to get the difference image and the histogram corresponding to the difference image. The initial key $x_{2}\left(0\right)$ is processed in the same way, as shown in Figure 7. By adding $10^{-3}$ to the initial key $x_{1}\left(0\right)$, four sequences are obtained through cellular neural chaos, and these four sequences are compared with the four sequences generated by no change of $x_{1}\left(0\right)$, as shown in Figure 8. It can be seen from Figure 7 and Figure 8 that the encryption system designed in this paper has high security and strong sensitivity to keys, which increases the difficulty for attackers to decipher the cipher image.

Plaintext sensitivity is also one of the important indexes of encryption system security, which indicates the ability of encryption system to resist the differential attack. A secure encryption system should be highly sensitive to plain image. The Number of Pixels Change Rate (NPCR) and Unified Average Changing Intensity (UACI) can be used to represent the difference between two plain images with one pixel difference. The calculation formula is [56]:

(13) $$\left\{\begin{array}{c} NPCR=\frac{1}{H\times W}\times \sum_{i=1}^{H}\sum_{j=1}^{W}D(i,j)\times 100\%\\ UACI=\frac{1}{H\times W}\times \sum_{i=1}^{H}\sum_{j=1}^{W}\frac{\left|v_{1}\left(i,j\right)-v_{2}\left(i,j\right)\right|}{255}\times 100\% \end{array}\right.$$

where $D\left(i,j\right)=\left\{\begin{array}{c} 0,v_{1}\left(i,j\right)=v_{2}\left(i,j\right)\\ 1,v_{1}\left(i,j\right)\neq v_{2}\left(i,j\right) \end{array}\right.$. $v_{1}\left(i,j\right)$ and $v_{2}\left(i,j\right)$ denote the pixel values at positions $v_{1}$ and $v_{2}$. For a digital image with a gray level of 256, 99.6094% and 33.4635% are ideal values of the NPCR and UACI, respectively.

Firstly, select a pixel from the “Lena” gray image randomly so that we can obtain a new image by changing its pixel value. Then, the two gray images which differ by only one pixel are each encrypted to obtain two ciphertext images. Finally, the NPCR and UACI values of the two encrypted images are obtained and the above operations will be repeated 50 times to obtain 50 groups of NPCR and UACI values. The NPCR and UACI average values of the gray images are shown in Table 3.

**Figure 9:**
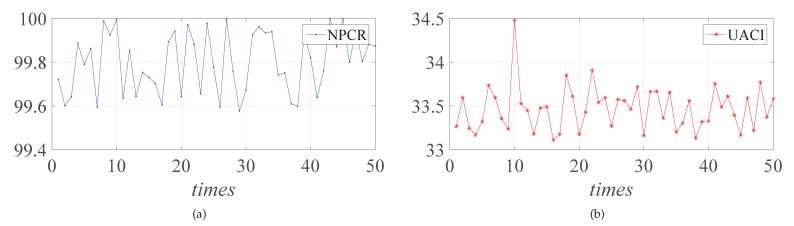
NPCR (**a**) and UACI (**b**).

The NPCR and UACI values obtained each time are shown in Figure 9. The NPCR and UACI average values are very close to the theoretical value. Therefore, the encryption system designed in this paper is extremely sensitive to both plain images and keys. The encryption algorithm designed in this study is safer and can resist the differential attack.

### 4.6. Information Entropy Analysis

The degree of the randomness of the system can be expressed by information entropy. The information entropy of the image is positively correlated with the encryption effect. The larger the information entropy is, the better effect the encryption will have. The formula of information entropy is defined as [57]:

(14) $$H\left(n\right)=-\sum_{i=0}^{G-1}-1P\left(n_{i}\right)\log_{2}P\left(n_{i}\right)$$

where *G* represents the number of gray level values of the image and $P(n_{i})$ the frequency of pixels with gray value *i*. The range of gray value of an image with a gray level of 256 is [0,255], and 8 is its ideal information entropy. When the value of information entropy is closer to 8, the image encryption has better effect [58].

Table 4 shows the information entropy before and after image encryption. The information entropy of the cipher image is very close to the theoretical value of information entropy. It is proven that the pixel value distribution of the cipher image is highly random and the encryption effect is better. Therefore, the algorithm can effectively resist the information entropy attack [33].

### 4.7. Psnr and Ssim

Peak Signal-to-Noise Ratio (PSNR) and Structural SIMilarity (SSIM) are often used to reflect the encryption quality. PSNR is essentially the same as the Mean Square Error (MSE) and can be obtained by MSE. The calculation formula is [59]:

(15) $$\left\{\begin{array}{c} MSE=\frac{1}{H\times W}\sum_{i=1}^{H}\sum_{j=1}^{W}{\left(P\left(i,j\right)-C\left(i,j\right)\right)}^{2}\\ PSNR=10\times \log_{10}\left(\frac{Q^{2}}{MSE}\right) \end{array}\right.$$

where the height and width of the image are represented by *H* and *W*, respectively, the pixel level of the image is represented by *Q*, the plain image pixels are represented by P(i,j), and the cipher image pixels are represented by C(i,j). SSIM is defined as [59]:

(16) $$SSIM\left(p,c\right)\;=\;\frac{\left(2\mu_{p}\mu_{c}\;+\;{\left(0.01L\right)}^{2}\right)\;\left(2\sigma_{pc}\;+\;{\left(0.03L\right)}^{2}\right)}{\left(u_{p}^{2}\;+\;u_{c}^{2}\;+\;{\left(0.01L\right)}^{2}\right)\left(\sigma_{p}^{2}\;+\;\sigma_{c}^{2}\;+\;{\left(0.03L\right)}^{2}\right)}$$

where the average values of the plain image *P* and the cipher image *C* are denoted by $u_{p}$ and $u_{c}$, respectively. The variance of the plain image and the cipher image denoted by $\sigma_{p}^{2}$ and $\sigma_{c}^{2}$ indicates that the covariance of the plain image and the cipher image represented by $\sigma_{pc}$. ${\left(0.01L\right)}^{2}$ and ${\left(0.03L\right)}^{2}$ are used as constant numbers to maintain stability. *L* represents the dynamic range of pixel values.

The range of SSIM is from −1 to 1. When the two images are the same, SSIM is 1. The smaller the PSNR and SSIM are, the better the encryption quality is. Table 5 and Table 6 show the encryption quality of the proposed scheme and the classic encryption schemes in recent years.

The experimental results show that the PSNR and SSIM values obtained by the proposed algorithm are lower than those of other proposed approaches. Therefore, this encryption scheme has certain advantages, and the image encryption quality is high.

### 4.8. Robust Noise Analysis

Robustness means that the system still has certain performance under interference or at random. Image robustness refers to the fact that the image still has a certain degree of fidelity after undergoing various signal processing or attacks. The image can still be recognized, with low distortion. Add 20% salt-and-pepper noise and 80×80 occlusion noise to the cipher image “Figure 5a”. The experimental results are shown in the figure below [34,60,61].

**Figure 10:**
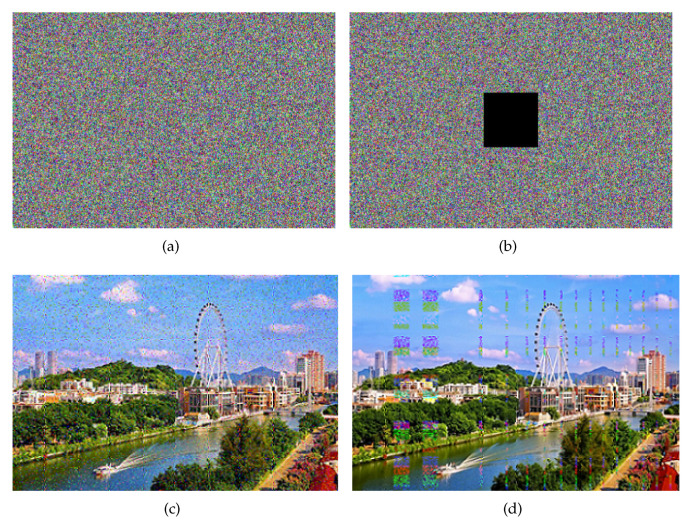
(**a**) Salt-and-pepper noise cipher image; (**b**) Occlusion noise cipher image; (**c**) Decryption of cipher image with salt-and-pepper noise; (**d**) Decryption of cipher image with occlusion noise.

It can be seen from Figure 10 that the decrypted images can still be easily identified with high fidelity after noise is added to the cipher image, which indicates the robustness of the image encryption system that can resist noise attacks.

## 5. Conclusions

This paper proposes a security-enhanced image communication scheme based on CNN under the cryptanalysis. First, the complex characteristics of CNN are used to generate some sequences. Then, a plain image and these CNN-based sequences are confused, permuted and diffused to get the cipher image. Utilizing the complex dynamics of CNN can effectively enhance the confusion, diffusion and avalanche of encryption. Theoretical analysis and experimental results both demonstrate its safety performance. From the perspective of cryptanalysis, the structure of an image cipher can effectively resist various common attacks. Therefore, the image communication scheme based on CNN proposed in this paper is a competitive security technology method.

## Figures and Tables

**Table 1:** NIST-800-22 test results.

Statistical Tests	*p*-Values										Result
	Seq1	Seq2	Seq3	Seq4	Seq5	Seq6	Seq7	Seq8	Seq9	Seq10	
ApproximateEntropy Text	0.8094	0.1941	0.0781	0.3518	0.4390	0.3812	0.4203	0.1690	0.1884	0.0589	Successful
BlockFrequency Text	0.9347	0.2822	0.9547	0.0925	0.6961	0.4518	0.1352	0.4160	0.3816	0.1934	Successful
CumulativeSums Text-1	0.7034	0.9290	0.7701	0.4770	0.0354	0.6270	0.4488	0.2083	0.4378	0.5493	Successful
CumulativeSums Text-2	0.8561	0.9968	0.8754	0.7377	0.0426	0.2912	0.2621	0.1019	0.3783	0.1853	Successful
FFT Text	0.9732	0.9066	0.4508	0.2911	0.4921	0.1912	0.8145	0.4508	0.0226	0.1359	Successful
Frequency Text	0.8666	0.8408	0.9040	0.4541	0.0235	0.6507	0.7674	0.1743	0.9330	0.5541	Successful
LinearComplexity Text	0.2833	0.8136	0.5262	0.2415	0.6749	0.4776	0.9849	0.2676	0.8014	0.3305	Successful
LongestRun Text	0.3615	0.2823	0.5065	0.4150	0.7894	0.7386	0.0683	0.1561	0.5800	0.2138	Successful
OverlappingTemplate Text	0.2713	0.8537	0.8457	0.6464	0.2555	0.1803	0.4144	0.9091	0.7819	0.7349	Successful
Rank Text	0.6985	0.1675	0.6198	0.2927	0.5757	0.3860	0.3147	0.8761	0.3737	0.2093	Successful
Runs Text	0.6066	0.6691	0.6771	0.2721	0.3432	0.1041	0.5789	0.7783	0.6718	0.6011	Successful
Serial Text-1	0.0096	0.8837	0.0110	0.5441	0.1669	0.0331	0.8454	0.1955	0.7045	0.6886	Successful
Serial Text-2	0.1784	0.6697	0.2170	0.5832	0.0293	0.3877	0.9621	0.4920	0.7287	0.5582	Successful

**Table 2:** Correlation coefficients of two adjacent pixels.

Pictures	Plain Image				Cipher Image			
	Vert.	Horiz.	Diag.	Anti-Diag.	Vert.	Horiz.	Diag.	Anti-Diag.
7.1.02.tiff	0.9480	0.9429	0.9113	0.9456	−0.0021	0.0303	0.0087	−0.0002
7.1.09.tiff	0.9309	0.9654	0.9208	0.9207	−0.0083	−0.0257	−0.0354	−0.0225
5.1.12.tiff	0.9709	0.9608	0.9429	0.9403	−0.0256	−0.0035	0.0040	−0.0157
5.2.10.tiff	0.9415	0.9364	0.9032	0.9015	0.0032	0.0163	−0.0069	−0.0107

**Table 3:** NPCR and UACI.

Pictures	NPCR (99.6094%)	UACI (33.4635%)
1.2.04.tiff	99.6093%	33.5974%
1.2.07.tiff	99.6078%	33.5580%
1.2.08.tiff	99.6154%	33.5209%
5.1.11.tiff	99.5544%	33.4018%

**Table 4:** Information entropy of the plain image and cipher image.

Pictures	Plain Image	Cipher Image
7.1.02.tiff	4.0045	7.9993
5.1.11.tiff	6.4523	7.9970
5.1.12.tiff	6.7057	7.9972
5.2.10.tiff	5.7056	7.9992

**Table 5:** PSNR of cipher image with different algorithms.

Pictures	This Paper	Ref. [1]	Ref. [60]	Ref. [28]
7.1.02.tiff	8.9518	9.1033	8.9731	8.9801
5.2.10.tiff	8.7620	8.7684	8.7660	8.7621
5.1.13.tiff	4.9032	4.9585	4.9168	4.9141
5.2.08.tiff	9.6225	9.6389	9.6378	9.6198

**Table 6:** SSIM of cipher image based on different algorithms.

Pictures	This Paper	Ref. [1]	Ref. [60]	Ref. [28]
7.1.02.tiff	0.0102	0.0108	0.0103	0.0109
5.1.11.tiff	0.0101	0.0099	0.0101	0.0109
5.2.10.tiff	0.0087	0.0098	0.0100	0.0091
5.1.13.tiff	0.0037	0.0057	0.0085	0.0067

## Data Availability

Not applicable.
